# Identifying Common Genes and Pathways Associated with Periodontitis and Aging by Bioinformatics Analysis

**DOI:** 10.1155/2022/4199440

**Published:** 2022-11-17

**Authors:** Qi Xie, Hongyu Lv, Tianqi Wang, Jingxuan Sun, Yuekun Li, Yumei Niu, Weili Xie

**Affiliations:** ^1^Department of Endodontics, The First Affiliated Hospital of Harbin Medical University, Harbin, Heilongjiang 150001, China; ^2^Department of Stomatology, Harbin Children's Hospital, Harbin, Heilongjiang 150001, China; ^3^Department of Prosthodontics, The First Affiliated Hospital of Harbin Medical University, Harbin, Heilongjiang 150001, China

## Abstract

**Background:**

This work used bioinformatic analysis to identify the relationship between periodontitis (PD) and aging, which could lead to new treatments for periodontal disease in the elderly.

**Method:**

Four microarray datasets were obtained from the Gene Expression Omnibus (GEO) database and analyzed in R language to identify differentially expressed genes (DEGs). The common DEGs of PD and aging were evaluated as key genes in this investigation by a Venn diagram. These common DEGs were analyzed through additional experiments and analysis, such as pathway analysis and enrichment analysis, and a network of protein-protein interactions (PPIs) was constructed. Cytoscape was used to visualize hub genes and critical modules based on the PPI network. Interaction of TF-genes and miRNAs with hub genes is identified.

**Result:**

84 common DEGs were found between PD and aging. Cytohubba was performed on the PPI network obtained from STRING tool, and the top 10 genes (MMP2, PDGFRB, CTGF, CD34, CXCL12, VIM, IL2RG, ACTA2, COL4A2, and TAGLN) were selected as hub genes. VIM may be a potential biomarker in the analysis of linked hub gene regulatory networks, and hsa-mir-21 and hsa-mir-125b are predicted to be associated in PD and aging.

**Conclusion:**

This study investigated the key genes and pathways interactions between PD and aging, which may help reveal the correlation between PD and aging. The current research results are obtained by prediction, and follow-up biological experiments are required for further verification.

## 1. Introduction

Periodontitis, an infectious and chronic inflammatory disease, is one of the world's most common chronic diseases, affecting roughly 11% of adults globally [1, 2]. Without proper treatment, PD may cause damage to the periodontal soft and hard tissues, leading to the formation of periodontal pockets, loss of attachment, resorption of alveolar bone, and ultimately tooth loss [3].

Aging is a time-dependent process characterized by the progressive deterioration and loss of function of multiple organs of an organism and was recently introduced by the World Health Organization as a new disease in the International Classification of Diseases [4]. From a biological point of view, with age, many molecular pathways are deregulated due to the accumulation of various molecules and cellular damage, thereby affecting tissue homeostasis, causing structural damage, and affecting its biomechanical properties [5, 6].

In recent years, an increasing number of studies have found that PD is closely linked to the advancement of aging-related systemic disorders such as diabetes, coronary heart disease, and rheumatoid arthritis [7–9]. Although aging itself has not been classified as a contributing factor to periodontitis [10], recent studies have shown that similar to other systemic diseases, PD susceptibility changes with aging, and the degree of alveolar bone destruction is related to age factors [11]. The reason may be that one of the major changes occurring in the aging process is the dysregulation of the immune response, leading to the dysregulation of the proinflammatory mediators and the chronic systemic inflammatory state of [12]. However, the hyperactivation of the immune response in the host system can directly activate the osteoclast activity and the alveolar bone loss, which can further induce the progression of periodontal disease [13]. However, the underlying mechanisms between PD and aging are not fully understood. As a result, additional research on the function of aging in PD will contribute to a better understanding and treatment of periodontal disease in the elderly. It may provide new ideas for the study of aging-related systemic disorders as well as prospective novel tactics for the treatment of periodontal diseases in the elderly.

**Figure 1:**
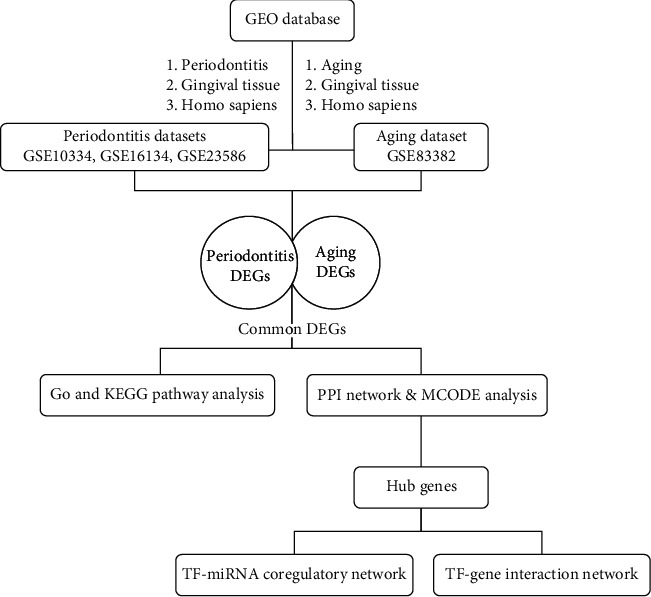
Workflow chart of the study.

To address this question, we employed an integrative bioinformatics approach to identify common functional genes associated with PD and aging. Moreover, investigation of DEGs in four datasets from the GEO can discover common mechanisms of action and PPI nodes. It may provide a basic theoretical basis for the diagnosis and pathogenesis of periodontal disease in the elderly.  Figure 1 depicted the progressive workflow of our study.

## 2. Materials and Methods

### 2.1. Data Sources

GEO (https://www.ncbi.nlm.nih.gov/geo) is a public database developed and maintained by the National Center for Biotechnology Information (NCBI), which stores gene expression data, microarrays, and other forms of high-throughput functional genomic data and makes it available to researchers for free [14]. This study retrieved the gene expression profiling datasets GSE10334, GSE16134, GSE23586, and GSE83382 from the GEO, based on the keywords “periodontitis and gingival tissue,” “aging and gingival tissue,” and “Homo sapiens.” These datasets were screened based on inclusion criteria. The inclusion criteria were as follows: (1) interdental CAL detectable at ≥2 nonadjacent teeth or (2) buccal or oral with pocketing >3 mm detectable at ≥2 teeth. Details of the microarray dataset are presented in Table 1.

### 2.2. DEG Identification

The primary goal of this work is to identify common DEGs from the GSE10334, GSE16134, GSE23586, and GSE83382 datasets. To identify the DEGs of GSE83382, we used the R language and the limma package (version 3.42.2) [15]. The GEO2R (https://www.ncbi.nlm.nih.gov/GEE351452r/) web tool, which also employs the limma program to detect DEGs, was used to evaluate the DEGs from the GSE10334, GSE16134, and GSE23586 datasets. GEO2R (version 3.26.8) is an online GEO analysis application that allows users to analyze and compare data from two or more separate sample groups under similar experimental settings [16]. DEGs were defined as genes with screening criteria *|*Log 2 fold change (FC)*|* > 0.5 and *P* value < 0.05. The intersection of common DEGs from the four datasets was defined as public DEGs in the following study and was found using the Venn diagram network tool (version 1.6.20) (http://bioinfogp.cnb.csic.es/tools/venny/), and finally, the primary experimental genes for the entire study were determined.

### 2.3. DEG Analysis at the Functional Level

Gene Ontology (GO) enrichment analysis is a technique for examining gene expression data. Enrichment is the process of classifying genes based on past knowledge, such as genome annotation information. GO is a method for identifying genes, gene products, and sequences as potential biological phenomena based on their roles in order to annotate and classify genes. It primarily consists of biological processes (BP), cellular components (CC), and molecular functions (MF) [17]. KEGG is a comprehensive database resource for biological interpretation of genomic sequences and other high-throughput data [18]. WebGestalt (http://www.webgestalt.org/), a long-standing and extensively used web program for functional enrichment analysis, has been regularly updated to fulfill the needs of biologists from various research fields [19]. WebGestalt (2019) was used in this study to perform GO and KEGG analysis on common DEGs. The usual measure was a *P* value of 0.05.

### 2.4. PPI Network Analysis

The search tool to retrieve interacting genes/proteins (STRING) (https://string-db.org/) is an interactive network for studying gene and protein interactions, supplementing heuristic association and analysis methods, and investigating known and predicted PPI associations with PD and aging. In this investigation, we used a string database to build a PPI network of DEGs and interacted with a composite score of >0.4 as a crucial criterion [20]. In addition, we used Cytoscape (https://cytoscape.org/) to build the PPI network for visual representation and additional experimental testing. Cytoscape (version 3.9.1) is a free and open-source network visualization platform that combines biomolecular interaction networks, high-throughput expression data, and other molecular states into a cohesive conceptual framework [21].

### 2.5. Hub Gene Extraction and Module Analysis

The PPI network is made up of nodes, edges, and connections, with the most entangled nodes serving as hub genes. Cytohubba (http://apps.cytoscape.org/apps/cytohubba) is a new Cytoscape plugin for sorting and retrieving central or possible or potential target elements underpinning biological networks. The cellular landscape plugin Cytohubba can discover hub genes more precisely thanks to 11 algorithms [22]. Furthermore, the Molecular Complex Detection (MCODE) (http://apps.cytoscape.org/apps/mcode) plugin Cytoscape was used with a plugin with default parameters to discriminate the modules that best reflect DEG clusters [23].

### 2.6. Transcription Factors and miRNAs Interact with Hub Genes

A network analysis database was used to investigate the human transcription factors (TFs) of the discovered hub genes. TF-gene interactions with identified hub genes evaluated TF outcomes at the functional pathway and gene expression levels [24]. NetworkAnalyst (https://www.networkanalyst.ca/) (version 3.0) is a complete platform of web tools that allows researchers to execute a wide range of common and difficult meta-analyses on gene expression data via an easy-to-use web interface [25]. In addition, miRNAs targeting gene interactions were incorporated, and TF-miRNA coregulated interactions were collected from a network library [26]. Finally, the Cytoscape tool was used to show the network of interactions between TFs, miRNAs, and hub genes.

## 3. Results

### 3.1. DEG Identification

**Figure 2:**
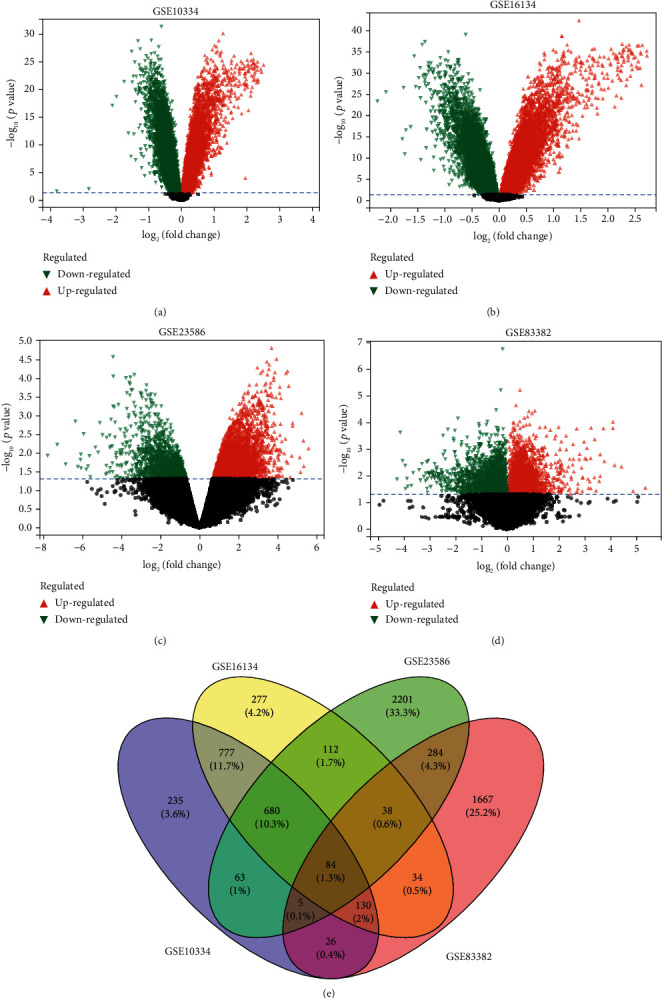
Identification of gene expression profiles in the four datasets. (a–c) Volcano plot of PD microarray data. (d) Volcano plot of aging microarray data. (e) Venn diagram of the 84 common DEGs between PD and aging.

As described in the materials and methods section, we performed DEG analysis on the screened samples and used volcano plots to show the final screening results of DEGs (Figures 2(a)–2(d)). We identified 2000 DEGs in GSE10334, 2132 DEGs inGSE16134, 3467 DEGs in GSE23586, and 2268 DEGs in GSE83382. Whereas for extraction, all significant DEGs are based on *|*Log 2 fold change (FC)*|* > 0.5 and *P* value < 0.05. A Venn diagram network tool was used to visualize common DEGs among the four datasets. These common genes were the main experimental gene for the entire study and were used to complete the next experiments. The Venn diagram revealed 84 shared differential genes (Figure 2(e)), including 24 coupregulated genes and 5 codownregulated genes.

### 3.2. DEG Enrichment Analysis by GO and KEGG Pathways

**Figure 3:**
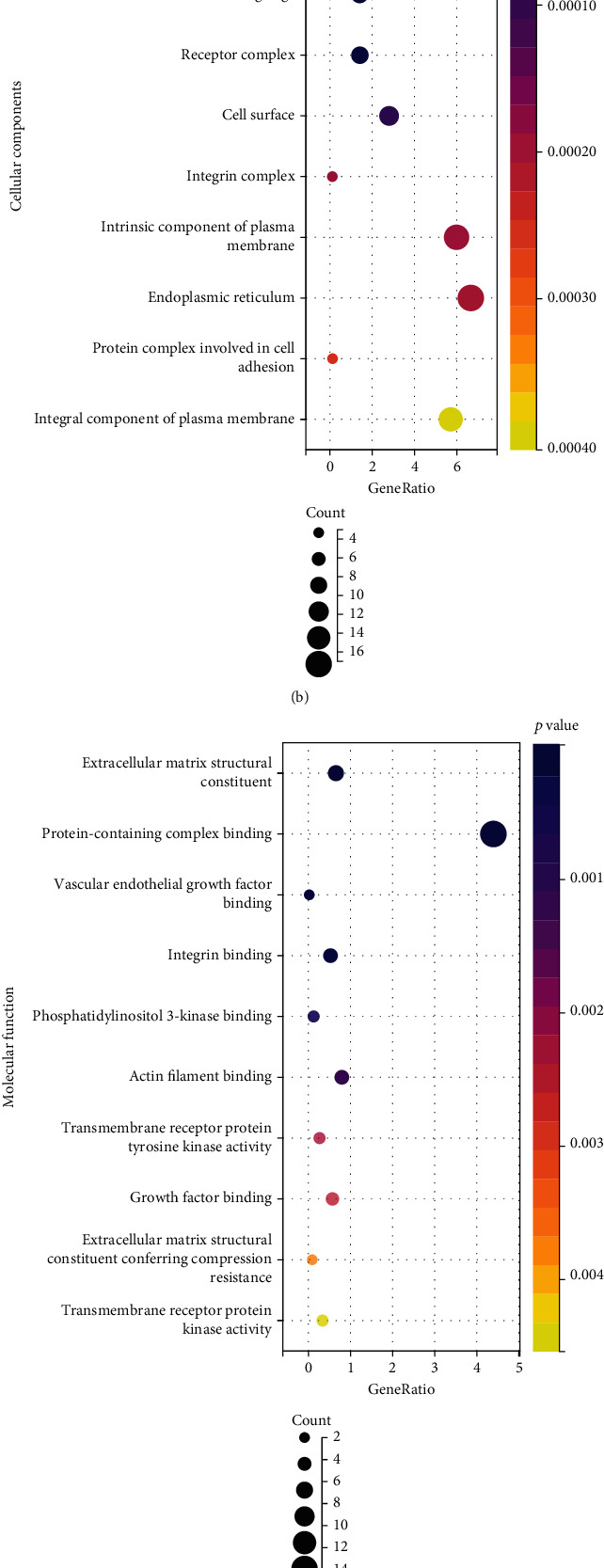
GO function enrichment analysis and KEGG pathway analysis of common DEGs. (a) Biological process. (b) Cell component. (c) Molecular function. (d) KEGG pathway.

Based on the screened common DEGs, we ran GO enrichment and KEGG pathway analysis to assess the biological processes, cellular components, and molecular function of gene functions and their linked pathways.  Figure 3 depicted the top ten substantially enriched phrases for each process with a linear connection in a histogram for each category. Extracellular structure organization, extracellular matrix organization, cell adhesion, biological adhesion, cell-substrate adhesion, blood vessel formation, blood vessel morphogenesis, and so on are all part of the BP category. These genes were abundant in collagen-containing extracellular matrix, extracellular matrix, cell leading edge, receptor complex, cell surface, and integrin complexes on the CC side. Extracellular matrix structural components, protein-containing complex binding, vascular endothelial growth factor binding, integrin binding, phosphatidylinositol 3-kinase binding, and actin filament binding were the most abundant genes in this category for MF. KEGG pathway analysis revealed that these genes were significantly involved in focal adhesion, PI3K-Akt signaling pathway, ECM-receptor interaction, regulation of actin cytoskeleton, leukocyte transendothelial migration, and cell adhesion molecules (CAMs).

### 3.3. PPI Network Identification Hub Genes and Module Analysis

**Figure 4:**
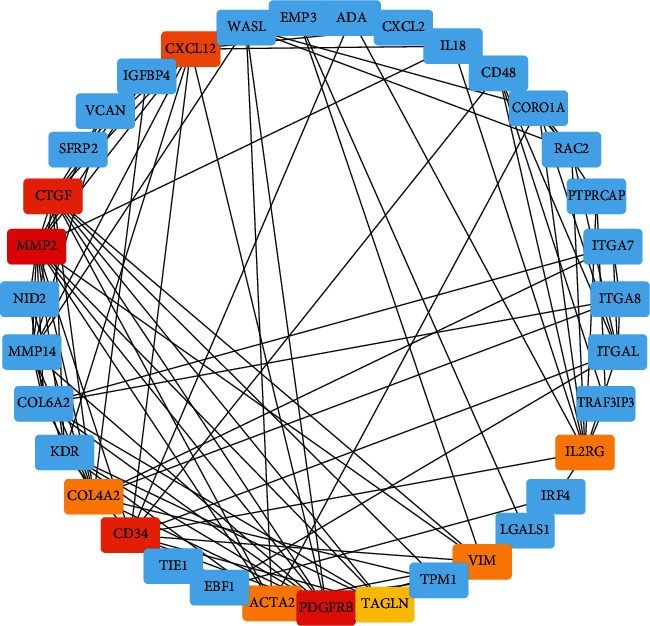
Detection of hub genes from the PPI network of common DEGs. The highlighted ten genes are hub genes.

To clarify the interactions between DEGs, we performed a PPI network analysis on all common DEGs in this study (Figure 4). The resulting PPI network contains 55 nodes and 133 edges. At the same time, we used Cytoscape to analyze the topological logic properties of the network. In the analysis results, we found highly connected genes, among which MMP2, PDGFRB, CTGF, CD34, CXCL12, VIM, IL2RG, ACTA2, COL4A2, and TAGLN were the top 10 highly connected genes (Supplementary Table 1). We then defined these 10 genes as hub genes. Subsequently, we reviewed the regulatory roles of these hub genes in PD and aging (Table 2).

**Figure 5:**
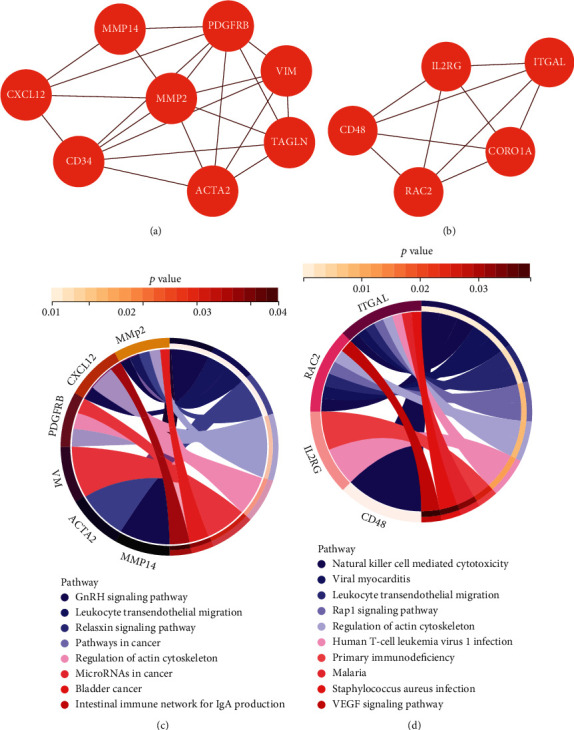
Two modules and enrichment analysis of PPI network. (a, b) Modules analysis network obtained from PPI network. (c, d) Functional annotation of modules.

The identified common DEGs were projected onto the Cytoscape MCODE plugin to assess key modules. The two most critical modules, which include the specified hub genes, are chosen from among all the modules. We used KEGG pathway analysis on the chosen modules and discovered that the genes in the two modules were considerably significantly enriched in the GnRH signaling route, leukocyte transendothelial migration, relaxin signaling pathway, cancer pathways, and actin cytoskeleton regulation (Figure 5).

### 3.4. TF-Gene Interaction Network

**Figure 6:**
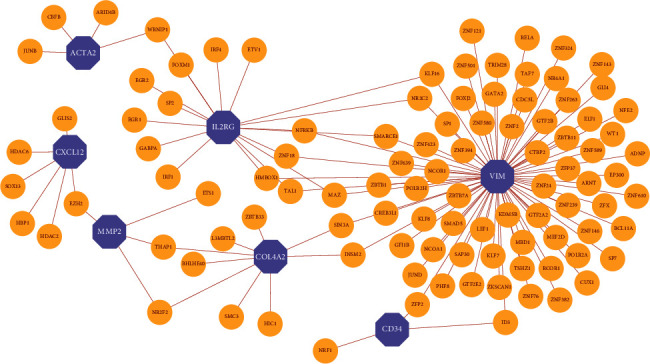
Construction of TF-hub gene interaction network from Cytoscape. The purple color octagons represent the hub genes, and the yellow nodes represent TF-genes.

We examined the connections between hub genes to discover significant changes occurring at the transcriptional level and obtain insight into the most critical regulatory molecules among hub genes. NetworkAnalyst network analysis program found 109 nodes and 118 edges of 7 hub genes. The generated data were then put into Cytoscape software to visualize the interactions between TFs and hub genes (Figure 6). The expression values and characteristics of hub genes in individual cohorts are shown in Supplementary Table 2. The results showed that VIM was regulated by 75-TF genes, IL2RG was regulated by 17 TF-genes, and COL4A2 was regulated by 9-TF genes, which indicated that the above three genes may be key genes in the TF-target network of PD and aging.

### 3.5. TF-miRNA Coregulatory Network

**Figure 7:**
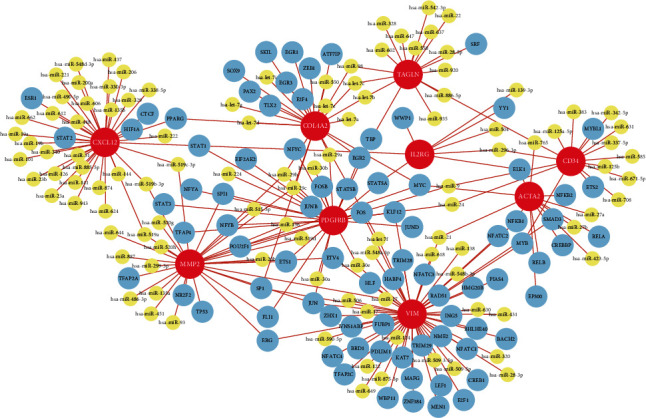
The network presents the TF-miRNA coregulatory network. The nodes in red color are the hub genes, yellow nodes represent miRNAs, and blue nodes indicate TF-genes.

A network analysis framework was used to build TF-miRNA coregulatory networks. The TF-miRNA coregulatory network splits TF-miRNA interactions and hub genes. This connection might be in charge of controlling hub gene expression. The TF-miRNA coregulation network was constructed with 204 nodes and 238 edges. Hub genes were interacted with by 109 miRNAs and 85 TF-genes (Figure 7). The expression values and characteristics of hub genes in individual cohorts are shown in Supplementary Table 3.

## 4. Discussion

With the global aging and the increase in the number of remaining teeth in the oral cavity, the demand for periodontal disease treatment and health care for the elderly population will continue to grow. A great number of studies have linked PD to age-related systemic disorders such as coronary heart disease and rheumatoid arthritis, indicating that chronic periodontal inflammation may increase with the aging of tissues and organs. Therefore, exploring the impact of aging on PD and its mechanism of action is critical [44, 45].

As an emerging interdisciplinary subject, bioinformatics has been widely used in the field of life sciences. Recently, the use of bioinformatics tools to study the genetic relationship between aging and inflammatory diseases, studies have found some correlation in molecular pathogenesis and disease development [46–48]. In this study, the four datasets identified 84 common DEGs, including 24 common upregulated genes and 5 common downregulated genes. DEGs were subjected to GO functional enrichment analysis and KEGG pathway analysis at the same time. The protein interaction network and the regulatory network between the proteins expressed by the differential genes were visualized and studied, and 10 hub genes were screened out, including MMP2, PDGFRB, CTGF, CD34, CXCL12, VIM, IL2RG, ACTA2, COL4A2, and TAGLN.

By performing GO functional enrichment analysis on DEGs, GO items were selected according to the *P* value. For BP, it mainly focuses on extracellular structural organization, extracellular matrix organization, cell adhesion bioadhesion, cell-substrate adhesion, vascular development, and vascular morphogenesis. In vitro, it enhanced the secretion of proinflammatory cytokines by macrophages by upregulating the expression of endothelial cell adhesion molecules [49]. In the CC, vascular development and vascular calcification have long been recognized as a degenerative age-related pathology caused by passive deposition of extracellular matrix [50]. The most significant MF of GO is extracellular matrix structural constituent, protein-containing complex binding, vascular endothelial growth factor binding, integrin binding, phosphatidylinositol 3-kinase binding, and actin filament binding. Oral fibroblast aging has been discovered to affect ECM synthesis and, more crucially, the structure of ECM fibers, which may hinder wound healing in the elderly [51]. According to cellular composition, the top GO keywords are collagen-containing extracellular matrix, extracellular matrix, and cell leading edge. Cell sheets made from PDLSCs from elderly donors have lower osteogenic potential than cell sheets made from PDLSCs from young donors based on the synthesis of ECM proteins such as fibronectin, integrin 1, and collagen type I [52].

The investigation of KEGG pathways for common DEGs revealed that these DEGs were mostly engaged in the focal adhesion signaling route, the PI3K-Akt signaling pathway, and the ECM receptor interaction signaling system. Previous research has linked the focal adhesion signaling pathway and PI3K-Akt signaling to age-related diseases. It was observed that the PI3K-Akt-mTOR signaling pathway is active during hippocampus aging. Adhesion sites are made up of adhesion proteins such as integrins and extracellular proteins that attach cells to the ECM [53]. The PI3K-Akt signaling pathway is a pathway with extensive intracellular effects. PI3K mainly promotes cell survival and resists apoptosis by activating Akt. After activation of Akt signaling, it can inhibit a variety of proapoptotic factors [54]. Studies have shown that antiaging drugs play a balance between antiaging effects and oxidative stress by activating the PI3K-Akt signaling pathway [55].

In this study, two important modules of common DEGs were screened for KEGG signaling pathway analysis. The results showed that the modules are mainly related to GnRH signaling pathway, leukocyte transendothelial migration, relaxin signaling pathway, and other pathways and have high correlation with the occurrence of aging and the development of inflammation, which deserve further study [56].

To find the most important regulatory molecules among the 10 hub genes, we analyzed the correlation between the hub genes and constructed a regulatory network between the hub genes and the tf-genes and the miRNA. According to our analyses, most hub genes exhibited strong correlations, and VIM had direct or indirect connections with other genes in the TF target regulatory network. VIM stands for vimentin, which is a class III intermediate filament protein present in nonepithelial cells, notably mesenchymal cells, and plays a crucial role in intracellular structural stability. VIM, a biomarker of epithelial-to-mesenchymal transition, has also been used as a diagnostic, prognostic, and therapeutic marker for fibrotic diseases [57, 58]. VIM is associated with abilities responsible for signal transduction and kinase interactions, thereby exerting control over gene regulatory networks. According to its role in fibrosis, VIM expression is also regulated by TGF and pro-inflammatory cytokines [59].

The miRNAs regulate the expression of many genes in cells, so abnormal expression of miRNAs may have an impact on the development of diseases. By examining the interaction between hub genes, TFs and miRNAs, we discovered that several miRNAs are implicated in PD (such as hsa-mir-17, hsa-mir-21, hsa-mir-29a, hsa-mir-29b, hsa-mir-29c, hsa-mir-31, hsa-mir-125a-5p, and hsa-mir-125b)[60–63]and aging (such as hsa-mir-23a, hsa-mir-125b, hsa-mir-21, hsa-mir-137, and hsa-mir-144) [64–66].

Several limitations in this study should be acknowledged. First, this analysis is based on existing datasets to identify common DEGs. The datasets are relatively small in size, and the obtained results may be biased, which requires subsequent external experiments to verify in order to obtain reliable conclusions. Second, hub gene screening is based on the degree of node identification, and the results are purely hypothetical. Furthermore, functional studies are necessary to confirm the hub genes in PD and aging. Therefore, in future studies, we may provide a theoretical framework and basis based on existing research to explore the relationship between PD and aging through gain or loss of function on biological models.

## 5. Conclusion

In the present work, a new mechanism was proposed which explain that progression in pathogenesis of both diseases might due to the common hub genes that disturbs the pathways which ultimately leads to disease condition. Moreover, it was further found that the signaling systems such as adhesion signaling pathway, PI3K-Akt signaling pathway, and ECM receptor interaction may be mainly involved. Additionally, VIM may be a potential biomarker in the analysis of linked hub gene regulatory networks, and hsa-mir-21 and hsa-mir-125b are predicted to be associated in PD and aging. These findings may lay the theoretical foundation for future studies.

## Figures and Tables

**Table 1:** List of datasets used in this study.

Datasets	Organism	Sample	Platform	Control	Affected
GSE10334	Homo sapiens	Gingival tissue	GPL570	64	183
GSE16134	Homo sapiens	Gingival tissue	GPL570	69	241
GSE23586	Homo sapiens	Gingival tissue	GPL570	3	3
GSE83382	Homo sapiens	Gingival tissue	GPL11154	3	3

**Table 2:** Review the regulatory role of hub genes in PD and aging.

Genes	PD	Aging
MMP2	Compared with healthy individuals (controls), metalloproteases (MMP-2, MMP-9) cascade in the initiation and progression of inflammatory bone resorption and periodontal soft tissue destruction in patients with periodontitis. [27].	Skin aging with age or premature aging caused by light promotes cellular inflammation, ROS production, and the increase of hydrogen peroxide, and matrix metalloproteinase (MMP2) expression is upregulated. [28].

PDGFRB	PDGF receptor- (PDGFR-) *β* on periodontal cells is a crucial element for various functions, such as wound healing in periodontal tissue, and their expression decreases when subjected to fluid shear stress [29].	Oxidative stress-induced senescent vascular smooth muscle cells were obviously desensitized to stimulation by platelet-derived growth factor- (PDGF-) BB, which may have been caused by suppression of promoter activity, transcription, translation, and activation levels of PDGF receptor- (PDGFR-) *β* [30].

CTGF	CTGF promotes the fusion of preosteoclasts by downregulating Bcl6 and subsequently increasing the expression of dendritic cell-specific transmembrane protein in periodontitis [31].	An upregulation of CTGF expression has been demonstrated in senescent cells [32].

CD34	The highest concentration of CD34+ cells was observed in the group of patients with advanced periodontal disease [33]. Treatment of periodontitis has neutral effects on peripheral endothelial function but significantly decreases circulating CD34(+) cell count [34].	The absolute number of circulating CD34(+) cells progressively and significantly decreased with advancing age [35].

CXCL12	CXCL12 (SDF-1alpha) may be involved in the immune defense pathway activated during periodontal disease. Upon the development of diseased tissues, CXCL12 (SDF-1alpha) levels increase and may recruit host defensive cells into sites of inflammation [36].	CXCR4 pathway stimulated by CXCL12 regulated AKT activation, CREB phosphorylation, and P53 level to affect the process of aging and Alzheimer's disease [37].

VIM	VIM expression is also regulated by TGF and proinflammatory cytokines. VIM expression increased significantly as periodontitis developed severe [38].	It was reported that VIM expression in was significantly increased in aged skin fibroblasts [39].

ACTA2	None	Seven aging molecular phenotype-relevant key genes (ACTA2, CALD1, LMOD1, MYH11, MYL9, MYLK, and TAGLN) were identified, which were specifically upregulated in tumors and in relation to dismal prognosis [40].

COL4A2	COL4A2 in the ECM promotes osteogenic differentiation of PDLSCs through negative regulation of the Wnt/*β*-catenin pathway, which can be used as a potential therapeutic strategy to repair bone defects [41].	Aging suppresses the expression levels of Col4a1 and Col4a2 and affects basement membrane-related factors in the steady state [42].

TAGLN	None	Seven aging molecular phenotype-relevant key genes (ACTA2, CALD1, LMOD1, MYH11, MYL9, MYLK, and TAGLN) were identified, which were specifically upregulated in tumors and in relation to dismal prognosis [40].

IL2RG	None	The expression levels of IL7, IL2RG, and IL7R were significantly lower in the 90-year-old adults, as compared with the middle-aged offspring [43].

## Data Availability

The data used to support the findings of this study are available from the corresponding author upon reasonable request.

## Supplementary Materials

Supplementary Materials Supplementary Table 1: topological characteristics for top ten hub genes in PPI of DEGs. Supplementary Table 2: topological characteristics of hub genes in TF-gene interaction network. Supplementary Table 3: topological characteristics of hub genes in TF-miRNA coregulatory network.

## References

[1] Kinane D. F., Stathopoulou P. G., Papapanou P. N. (2017). Periodontal diseases. *Nature Reviews. Disease Primers*.

[2] Tonetti M. S., Jepsen S., Jin L., Otomo-Corgel J. (2017). Impact of the global burden of periodontal diseases on health, nutrition and wellbeing of mankind: a call for global action. *Journal of Clinical Periodontology*.

[3] Joshipura K. J., Muñoz-Torres F. J., Dye B. A., Leroux B. G., Ramírez-Vick M., Pérez C. M. (2018). Longitudinal association between periodontitis and development of diabetes. *Diabetes Research and Clinical Practice*.

[4] Khaltourina D., Matveyev Y., Alekseev A., Cortese F., Ioviţă A. (2020). Aging fits the disease criteria of the international classification of diseases. *Mechanisms of Ageing and Development*.

[5] Partridge L., Deelen J., Slagboom P. E. (2018). Facing up to the global challenges of ageing. *Nature*.

[6] Aquino-Martinez R., Eckhardt B. A., Rowsey J. L. (2021). Senescent cells exacerbate chronic inflammation and contribute to periodontal disease progression in old mice. *Journal of Periodontology*.

[7] Preshaw P. M., Alba A. L., Herrera D. (2012). Periodontitis and diabetes: a two-way relationship. *Diabetologia*.

[8] Nocini R., Favaloro E. J., Sanchis-Gomar F., Lippi G. (2020). Periodontitis, coronary heart disease and myocardial infarction: treat one, benefit all. *Blood Coagulation & Fibrinolysis: an International Journal in Haemostasis and Thrombosis*.

[9] Hussain S. B., Botelho J., Machado V. (2020). Is there a bidirectional association between rheumatoid arthritis and periodontitis? A systematic review and meta-analysis. *Seminars in Arthritis and Rheumatism*.

[10] Eke P. I., Dye B. A., Wei L. (2015). Update on prevalence of periodontitis in adults in the United States: NHANES 2009 to 2012. *Journal of Periodontology*.

[11] López R., Smith P. C., Göstemeyer G., Schwendicke F. (2017). Ageing, dental caries and periodontal diseases. *Journal of Clinical Periodontology*.

[12] Chung H. Y., Kim D. H., Lee E. K. (2019). Redefining chronic inflammation in aging and age-related diseases: proposal of the senoinflammation concept. *Aging and Disease*.

[13] Pan W., Wang Q., Chen Q. (2019). The cytokine network involved in the host immune response to periodontitis. *International Journal of Oral Science*.

[14] Edgar R., Domrachev M., Lash A. E. (2002). Gene expression omnibus: NCBI gene expression and hybridization array data repository. *Nucleic Acids Research*.

[15] Ritchie M. E., Phipson B., Wu D. (2015). Limma powers differential expression analyses for RNA-sequencing and microarray studies. *Nucleic Acids Research*.

[16] Barrett T., Wilhite S. E., Ledoux P. (2012). NCBI GEO: archive for functional genomics data sets--update. *Nucleic Acids Research*.

[17] Doms A., Schroeder M. (2005). GoPubMed: exploring PubMed with the gene ontology. *Nucleic Acids Research*.

[18] Kanehisa M., Goto S. (2000). KEGG: Kyoto encyclopedia of genes and genomes. *Nucleic Acids Research*.

[19] Wang J., Vasaikar S., Shi Z., Greer M., Zhang B. (2017). WebGestalt 2017: a more comprehensive, powerful, flexible and interactive gene set enrichment analysis toolkit. *Nucleic Acids Research*.

[20] Szklarczyk D., Franceschini A., Wyder S. (2015). STRING v10: protein-protein interaction networks, integrated over the tree of life. *Nucleic Acids Research*.

[21] Otasek D., Morris J. H., Bouças J., Pico A. R., Demchak B. (2019). Cytoscape automation: empowering workflow-based network analysis. *Genome Biology*.

[22] Chin C. H., Chen S. H., Wu H. H., Ho C. W., Ko M. T., Lin C. Y. (2014). cytoHubba: identifying hub objects and sub-networks from complex interactome. *BMC Systems Biology*.

[23] Bader G. D., Hogue C. W. (2003). An automated method for finding molecular complexes in large protein interaction networks. *BMC Bioinformatics*.

[24] Ye Z., Wang F., Yan F. (2019). Bioinformatic identification of candidate biomarkers and related transcription factors in nasopharyngeal carcinoma. *World Journal of Surgical Oncology*.

[25] Zhou G., Soufan O., Ewald J., Hancock R. E. W., Basu N., Xia J. (2019). NetworkAnalyst 3.0: a visual analytics platform for comprehensive gene expression profiling and meta-analysis. *Nucleic Acids Research*.

[26] Sethupathy P., Corda B., Hatzigeorgiou A. G. (2006). Tar Base: a comprehensive database of experimentally supported animal micro RNA targets. *Ribonucleic Acid*.

[27] Kluknavská J., Rabajdová M., Urban P. (2022). Expression of selected inflammatory proteins and metalloproteinases in periodontitis. *European Review for Medical and Pharmacological Sciences*.

[28] Xiao X., Huang M., Fan C., Zuo F. (2021). DUOX2 participates in skin aging induced by UVB in HSF2 cells by activating NF-*κ*B signaling. *Experimental and Therapeutic Medicine*.

[29] Zheng L., Shi Q., Na J., Liu N., Guo Y., Fan Y. (2019). Platelet-derived growth factor receptor-*α* and *β* are involved in fluid shear stress regulated cell migration in human periodontal ligament cells. *Cellular and Molecular Bioengineering*.

[30] Pan C. H., Chen C. J., Shih C. M., Wang M. F., Wang J. Y., Wu C. H. (2019). Oxidative stress-induced cellular senescence desensitizes cell growth and migration of vascular smooth muscle cells through down-regulation of platelet-derived growth factor receptor-beta. *Aging*.

[31] Choi Y., Yoo J. H., Lee J. H. (2020). Connective tissue growth factor (CTGF) regulates the fusion of osteoclast precursors by inhibiting Bcl 6 in periodontitis. *International Journal of Medical Sciences*.

[32] Kim K. H., Park G. T., Lim Y. B. (2004). Expression of connective tissue growth factor, a biomarker in senescence of human diploid fibroblasts, is up-regulated by a transforming growth factor-beta-mediated signaling pathway. *Biochemical and Biophysical Research Communications*.

[33] Aleksandrowicz P., Kotuła L. Z., Grabowska K. (2021). Evaluation of circulating CD34+ stem cells in peripheral venous blood in patients with varying degrees of periodontal disease. *Annals of Agricultural and Environmental Medicine: AAEM*.

[34] Li X., Tse H. F., Yiu K. H., Li L. S. W., Jin L. (2011). Effect of periodontal treatment on circulating CD34+ cells and peripheral vascular endothelial function: a randomized controlled trial. *Journal of Clinical Periodontology*.

[35] Moresi R., Tesei S., Costarelli L. (2005). Age- and gender-related alterations of the number and clonogenic capacity of circulating CD34+ progenitor cells. *Biogerontology*.

[36] Havens A. M., Chiu E., Taba M. (2008). Stromal-derived factor-1alpha (CXCL12) levels increase in periodontal disease. *Journal of Periodontology*.

[37] Li H., Hao L., Li Y., Wang R. (2018). Reducing CXCR4 resulted in impairing proliferation and promoting aging. *The Journal of Nutrition, Health & Aging*.

[38] Wadie K. W., Bashir M. H., Abbass M. M. S. (2021). Epithelial-mesenchymal transition in gingival tissues from chronic periodontitis patients: a case-control study. *Dental and Medical Problems*.

[39] Nishio K., Inoue A. (2005). Senescence-associated alterations of cytoskeleton: extraordinary production of vimentin that anchors cytoplasmic p 53 in senescent human fibroblasts. *Histochemistry and Cell Biology*.

[40] He F., Ding H., Zhou Y. (2021). Depiction of aging-based molecular phenotypes with diverse clinical prognosis and immunological features in gastric cancer. *Frontiers in Medicine*.

[41] Wen Y., Yang H., Wu J. (2019). COL4A2 in the tissue-specific extracellular matrix plays important role on osteogenic differentiation of periodontal ligament stem cells. *Theranostics*.

[42] Kanazawa Y., Nagano M., Koinuma S., Sugiyo S., Shigeyoshi Y. (2021). Effects of aging on basement membrane-related gene expression of the skeletal muscle in rats. *Biomedical Research (Tokyo, Japan)*.

[43] Passtoors W. M., van den Akker E. B., Deelen J. (2015). IL7R gene expression network associates with human healthy ageing. *Immunity & Ageing*.

[44] Carter C. J., France J., Crean S., Singhrao S. K. (2017). The porphyromonas gingivalis/host interactome shows enrichment in GWASdb genes related to Alzheimer's disease, diabetes and cardiovascular diseases. *Frontiers in Aging Neuroscience*.

[45] Persson G. R. (2018). Periodontal complications with age. *Periodontology, 2000*.

[46] Liu W., Brodsky A. S., Feng M. (2021). Senescent tissue-resident mesenchymal stromal cells are an internal source of inflammation in human osteoarthritic cartilage. *Frontiers in Cell and Developmental Biology*.

[47] Han X., Liu Y. J., Liu B. W., Ma Z. L., Xia T. J., Gu X. P. (2022). TREM2 and CD163 ameliorate microglia-mediated inflammatory environment in the aging brain. *Journal of Molecular Neuroscience: MN*.

[48] Blacher E., Tsai C., Litichevskiy L. (2022). Aging disrupts circadian gene regulation and function in macrophages. *Nature Immunology*.

[49] Suh J. S., Kim S., Boström K. I., Wang C. Y., Kim R. H., Park N. H. (2019). Periodontitis-induced systemic inflammation exacerbates atherosclerosis partly via endothelial-mesenchymal transition in mice. *International Journal of Oral Science*.

[50] Chao C. T., Yeh H. Y., Tsai Y. T. (2019). Natural and non-natural antioxidative compounds: potential candidates for treatment of vascular calcification. *Cell Death Discovery*.

[51] Atkuru S., Muniraj G., Sudhaharan T., Chiam K. H., Wright G. D., Sriram G. (2021). Cellular ageing of oral fibroblasts differentially modulates extracellular matrix organization. *Journal of Periodontal Research*.

[52] Wu R. X., Bi C. S., Yu Y., Zhang L. L., Chen F. M. (2015). Age-related decline in the matrix contents and functional properties of human periodontal ligament stem cell sheets. *Acta Biomaterialia*.

[53] Meng S., Xia W., Pan M., Jia Y., He Z., Ge W. (2020). Proteomics profiling and pathway analysis of hippocampal aging in rhesus monkeys. *BMC Neuroscience*.

[54] Vara J. Á. F., Casado E., de Castro J., Cejas P., Belda-Iniesta C., González-Barón M. (2004). PI3K/Akt signalling pathway and cancer. *Cancer Treatment Reviews*.

[55] Gong P., Wang D., Cui D. (2021). Anti-aging function and molecular mechanism of Radix Astragali and Radix Astragali preparata via network pharmacology and PI3K/Akt signaling pathway. *Phytomedicine*.

[56] Barabás K., Szabó-Meleg E., Ábrahám I. M. (2020). Effect of inflammation on female gonadotropin-releasing hormone (GnRH) neurons: mechanisms and consequences. *International Journal of Molecular Sciences*.

[57] Cardoso A. L., Fernandes A., Aguilar-Pimentel J. A. (2018). Towards frailty biomarkers: candidates from genes and pathways regulated in aging and age-related diseases. *Ageing Research Reviews*.

[58] Mendez M. G., Kojima S., Goldman R. D. (2010). Vimentin induces changes in cell shape, motility, and adhesion during the epithelial to mesenchymal transition. *FASEB Journal: Official Publication of the Federation of American Societies for Experimental Biology*.

[59] Mor-Vaknin N., Punturieri A., Sitwala K., Markovitz D. M. (2003). Vimentin is secreted by activated macrophages. *Nature Cell Biology*.

[60] Stoecklin-Wasmer C., Guarnieri P., Celenti R., Demmer R. T., Kebschull M., Papapanou P. N. (2012). MicroRNAs and their target genes in gingival tissues. *Journal of Dental Research*.

[61] Wa Lee Y. H., Am Na H. S., Jeong S. Y., Jeong S. H. E., Park H. R. Y., Chung J. (2011). Comparison of inflammatory microRNA expression in healthy and periodontitis tissues. *Biocell*.

[62] Xie Y. F., Shu R., Jiang S. Y., Liu D. L., Zhang X. L. (2011). Comparison of microRNA profiles of human periodontal diseased and healthy gingival tissues. *International Journal of Oral Science*.

[63] Jin S. H., Zhou J. G., Guan X. Y., Bai G. H., Liu J. G., Chen L. W. (2020). Development of an miRNA-array-based diagnostic signature for periodontitis. *Frontiers in Genetics*.

[64] Luo Z., Feng X., Wang H. (2015). mir-23a induces telomere dysfunction and cellular senescence by inhibiting TRF2 expression. *Aging Cell*.

[65] ElSharawy A., Keller A., Flachsbart F. (2012). Genome-wide miRNA signatures of human longevity. *Aging Cell*.

[66] Singhvi G., Manchanda P., Krishna Rapalli V., Kumar Dubey S., Gupta G., Dua K. (2018). MicroRNAs as biological regulators in skin disorders. *Biomedicine & Pharmacotherapy*.

