# Supplementary material for: Identifying Common Genes and Pathways Associated with Periodontitis and Aging by Bioinformatics Analysis
Source: Dis Markers. 2022 Nov 17;2022:4199440. doi: 10.1155/2022/4199440 (PMC9691312; doi:10.1155/2022/4199440)
Supplement: Supplementary Materials — Supplementary Table 1: topological characteristics for top ten hub genes in PPI of DEGs. Supplementary Table 2: topological characteristics of hub genes in TF-gene interaction network. Supplementary Table 3: topological characteristics of hub genes in TF-miRNA coregulatory network. [file 4199440.f1.docx]

**Supplementary Material**

Supplementary Table 1 Topological characteristics for top ten hub genes in PPI of DEGs

| Genes | Degree | Eccentricity | Closeness | Betweenness | Stress |
| --- | --- | --- | --- | --- | --- |
| MMP2 | 15 | 0.15455 | 29.31667 | 295.5797 | 1254 |
| PDGFRB | 13 | 0.18545 | 28.28333 | 180.713 | 900 |
| CTGF | 12 | 0.15455 | 26.81667 | 123.1696 | 516 |
| CD34 | 12 | 0.18545 | 28.45 | 424.1579 | 1546 |
| CXCL12 | 11 | 0.15455 | 26.23333 | 254.8413 | 994 |
| VIM | 10 | 0.15455 | 25.73333 | 237.174 | 738 |
| IL2RG | 10 | 0.18545 | 26.2 | 271.173 | 902 |
| ACTA2 | 10 | 0.18545 | 26.03333 | 116.4694 | 586 |
| COL4A2 | 10 | 0.15455 | 24.73333 | 111.3459 | 582 |
| TAGLN | 9 | 0.15455 | 25.23333 | 22.59449 | 126 |

Supplementary Table 2 Topological characteristics of hub genes in TF-gene interaction network

| Genes | Degree | Eccentricity | Closeness | Betweenness | Stress |
| --- | --- | --- | --- | --- | --- |
| VIM | 75.0 | 0.14286 | 84.54762 | 10770.0 | 41694.0 |
| IL2RG | 17.0 | 0.11111 | 44.56627 | 2634.0 | 26192.0 |
| COL4A2 | 9.0 | 0.14286 | 39.34524 | 3076.0 | 19324.0 |
| CXCL12 | 6.0 | 0.09091 | 21.47908 | 1050.0 | 7830.0 |
| ACTA2 | 4.0 | 0.09091 | 25.86129 | 636.0 | 6066.0 |
| MMP2 | 4.0 | 0.11111 | 26.07024 | 1615.0 | 12464.0 |
| CD34 | 3.0 | 0.11111 | 33.26151 | 215.0 | 958.0 |

Supplementary Table 3 Topological characteristics of hub genes in TF-miRNA coregulatory network

| Genes | Degree | Eccentricity | Closeness | Betweenness | Stress |
| --- | --- | --- | --- | --- | --- |
| VIM | 59.0 | 0.2 | 101.05 | 18347.89313 | 151902.0 |
| CXCL12 | 38.0 | 0.2 | 82.4 | 12515.17894 | 104870.0 |
| MMP2 | 31.0 | 0.2 | 85.18333 | 11638.47645 | 128992.0 |
| PDGFRB | 30.0 | 0.2 | 87.68333 | 11715.28771 | 83462.0 |
| COL4A2 | 26.0 | 0.2 | 83.7 | 9725.43421 | 55172.0 |
| TAGLN | 17.0 | 0.2 | 65.2 | 5096.97404 | 37074.0 |
| CD34 | 16.0 | 0.2 | 69.85 | 5040.48517 | 25816.0 |
| ACTA2 | 13.0 | 0.2 | 70.9 | 4254.89502 | 20386.0 |
| IL2RG | 8.0 | 0.2 | 60.26667 | 2419.37534 | 14886.0 |
